# Computational Models for Diagnosing and Treating Endometriosis

**DOI:** 10.3389/frph.2021.699133

**Published:** 2021-12-20

**Authors:** Wangui Mbuguiro, Adriana Noemi Gonzalez, Feilim Mac Gabhann

**Affiliations:** ^1^Department of Biomedical Engineering, Johns Hopkins University School of Medicine, Baltimore, MD, United States; ^2^Institute for Computational Medicine, Institute for NanoBioTechnology, Johns Hopkins University, Baltimore, MD, United States; ^3^Department of Biomedical Engineering, Johns Hopkins University, Baltimore, MD, United States

**Keywords:** endometriosis, hormone therapy, computational, machine learning, systems biology, mechanism, biomarker, pharmacokinetics

## Abstract

Endometriosis is a common but poorly understood disease. Symptoms can begin early in adolescence, with menarche, and can be debilitating. Despite this, people often suffer several years before being correctly diagnosed and adequately treated. Endometriosis involves the inappropriate growth of endometrial-like tissue (including epithelial cells, stromal fibroblasts, vascular cells, and immune cells) outside of the uterus. Computational models can aid in understanding the mechanisms by which immune, hormone, and vascular disruptions manifest in endometriosis and complicate treatment. In this review, we illustrate how three computational modeling approaches (regression, pharmacokinetics/pharmacodynamics, and quantitative systems pharmacology) have been used to improve the diagnosis and treatment of endometriosis. As we explore these approaches and their differing detail of biological mechanisms, we consider how each approach can answer different questions about endometriosis. We summarize the mathematics involved, and we use published examples of each approach to compare how researchers: (1) shape the scope of each model, (2) incorporate experimental and clinical data, and (3) generate clinically useful predictions and insight. Lastly, we discuss the benefits and limitations of each modeling approach and how we can combine these approaches to further understand, diagnose, and treat endometriosis.

## Introduction

### Endometriosis: A Complex Disease

Although observations of endometrial-like cells growing outside of the uterus were made as early as the nineteenth century (1), endometriosis remains a significant and understudied public health challenge. Endometriosis is estimated to afflict 10% of menstruators and 20–25% of women undergoing surgery due to infertility or pelvic pain (2, 3). One challenge to estimating this prevalence is the variability in endometriosis presentation—with some only discovering endometriosis incidentally during surgery and others living with a wide range of debilitating symptoms (4). People with symptomatic endometriosis suffer an average of 7 years before diagnosis, a delay exacerbated by the lack of a non-surgical diagnostic for the disease (5). There is no cure for endometriosis. Rather, those with suspected or diagnosed endometriosis must decide how to combine interventions that primarily address symptoms (e.g., hormonal contraceptives and hysterectomy) and those that target endometriosis lesions specifically (e.g., ablation or excision surgeries). Unfortunately, all of these interventions affect a patient's ability to conceive and have 5 year symptom recurrence rates ranging from 10 to 62% (6).

Endometriosis patients are typically staged by the visual appearance of lesions and adhesions according to the American Society for Reproductive Medicine's revised system. However, this staging does not correlate with patient symptoms or treatment outcomes (7). Looking beyond visual characteristics, clinical and experimental studies suggest that the growth and survival of lesions is enabled by a combination of immune dysfunction, hormone dysregulation, and aberrant blood vessel development (8–10). Specifically, endometriosis patients have been observed to have differences in progesterone receptor expression and functioning (11, 12), in peritoneal cytokine profiles, and in immune cell functioning (13, 14)—which all have the potential to interfere with the efficacy of pharmacological and surgical interventions.

To understand how such complex systems can contribute to patient symptoms and treatment outcomes, we need to integrate quantitative and computational approaches with clinical and experimental techniques. Researchers have created mathematical models to predict patient diagnoses and treatment outcomes based on symptoms, measurements, and medical history. However, as the success (and failure) of therapies is increasingly recognized as dependent on system-wide biological differences, computational models will need to expand in order to understand the mechanisms connecting these differences to clinical presentations and treatment outcomes.

In this review, we will first summarize how mathematical models have been used and modified over the years to study, diagnose, and treat endometriosis (in section “Systems Biology and Computational Models of Endometriosis”). We will then explore three mathematical modeling approaches to endometriosis that each take advantage of increasing detail in biological mechanisms. For each modeling approach, we will investigate their design, use of experimental and clinical data, and the insight they provide. Lastly, we will discuss current limitations in mathematical modeling of endometriosis and possible future directions in the conclusion section.

### Systems Biology and Computational Models of Endometriosis

**Systems biology** is an integrative approach to investigating how genetic, cellular, and tissue level differences can influence an organism's physiology. This could include using quantitative measurements, ranging from *in vitro* cell culture experiments to various clinical observations, to extensively characterize a biological system. These experimental and clinical data can then be analyzed using mathematical and computational modeling approaches to make predictions about how the biological system behaves under various conditions (Supplementary Table 1). But how do we represent this system complexity meaningfully in a model? There are several ways, with different levels of mechanistic detail.

#### Regression and Machine Learning

Early computational models of endometriosis to have impact on the clinic were **regression models** that helped develop non-surgical screening tools for endometriosis in symptomatic women [reviewed in (15)]. Regression is a form of **machine learning** and is primarily **data-driven**, basing predictions (e.g., the probability of a patient having endometriosis) on measurable characteristics (e.g., differences in age, weight, pain qualities, subfertility, etc.) without including any causal relationships. More advanced forms of regression modeling, such as mixed-effects modeling, have been used to identify symptom-based subtypes of endometriosis patients using electronic heath records (16) and patient self-reporting (16, 17). The findings of these models have aided in diagnosing endometriosis (discussed in section “Diagnosing Endometriosis—Regression Modeling”) and evaluating endometriosis treatment strategies (section “Gaps in Modeling Endometriosis and Opportunities for Future Models”).

With the advent of techniques to collect and analyze patient samples, researchers have identified possible biomarkers for endometriosis using measurements from the peritoneal fluid, blood, urine, eutopic endometrium, and more [reviewed in (18)]. Regression modeling has been used here to identify associations between endometriosis and gene expression regulators (19), cytokines, angiogenic factors, and growth factors (20). Additionally, other machine learning techniques have been used to identify and explore the significance of molecular abnormalities found in endometriosis (14, 21–23).

#### Mechanism-Based Modeling

In contrast to data-driven models, which base predictions on how biological *components* (e.g., patient features, protein levels, etc.) may be associated with a *phenomenon* (e.g., diagnosis or therapy response), **mechanism-based models** incorporate and attempt to understand the “how” in these associations. In other words, mechanism-based models use equations that reflect how *components* interact in *space* and *time* within a specific *context* (e.g., drug or antigen exposure) to affect said *phenomenon* (24) (Table 1). In applying a mechanism-based approach, systems biologists can synthesize experimental data from independent studies as they simulate experiments done in cell culture, animal experiments, and clinical trials. This has enabled the prediction of drug interactions, establishing the fields of quantitative systems pharmacology (28) and systems toxicology (29).

**Table 1:** Overview of mechanism-based modeling and discussion in this review.

**Defining feature of “mechanism”**	**Presence in regression modeling** **to diagnose endometriosis (25)**	**Presence in PK-PD modeling** **in treating endometriosis (26)**	**Presence in QSP modeling** **of menstrual cycle modulators (27)**
*Phenomenon*	Endometriosis diagnosis	Therapy delivery and effect on ovarian cyst formation	Therapy delivery and effect on ovulation
*Context*	Patients seeing clinicians for pain and/or infertility, without previous diagnosis	Patients receiving therapy (Intravaginal ring containing anastrozole and levonorgestrel)	Patients receiving therapy (Gonadotropin-releasing hormone analogs)
*Components*	Patient attributes (e.g., symptoms, characteristics, medical history) that may contribute to diagnosis	Patient attributes, drug, and endogenous molecules that affect response to therapy	Drug, cells, and endogenous molecules (e.g., hormones and receptors), that affect response to therapy
*Spatial arrangement*& *Temporal relationships*	(Not modeled)	Drug transport from intravaginal ring to non-specific body compartments	Synthesis, transport, and interactions between components throughout the hypothalamus, pituitary, and ovaries

*A biological mechanism includes all five features in the first column of this table [defined in (24)]*.

Early mechanism-based modeling relevant to endometriosis predicted ovarian follicle maturation in response to hormone cycling (30). Since then, several papers have expanded these models to predict the effects of exogenous hormones in people with normal menstrual cycles and in people with polycystic ovary syndrome [reviewed in (31)]. More recently, models with increasing levels of mechanistic detail have been developed to optimize hormonal therapies for treating endometriosis and other estrogen-associated conditions while minimizing adverse events [discussed in sections “Treating Endometriosis—Pharmacokinetic and Pharmacodynamic (PK-PD) Modeling” and “Modulating the Menstrual Cycle—Quantitative Systems Pharmacology (QSP) Models”].

#### Comparing Modeling Approaches (Scope, Data, Impact)

For all models, careful selection of scope is key—in other words, modelers choose which variables and parameters are included and which are not. What's included in the model will in turn affect how clinical and experimental data are used to create and validate the model. As a result, these modeling approaches will differ in the insight they can provide to clinical decisions and the impact this may have on patients. In this review, we compare how three computational studies design their model scope, use data, and impact clinical decisions.

## Diagnosing Endometriosis—Regression Modeling

### Motivation for Logistic Regression Modeling

The current “gold standard” for diagnosing endometriosis is laparoscopic surgery followed by histology to identify endometrial-like growths in the abdomen (5), but this surgery has several limitations that have led to it being commonly postponed or avoided. These include: its high cost, potential complications, and the need for a highly skilled endometriosis surgeon (32). Instead, blood tests, pelvic examinations, and ultrasound imaging are done to rule out other disorders. Of these methods, only ultrasound imaging can detect endometriosis; however, it is limited to only detecting one form of disease (ovarian endometriotic cysts) (20). As a result, researchers have turned to logistic regression to answer the question: Can a combination of clinical observations reliably predict endometriosis?

### Use of Logistic Regression Modeling to Guide Diagnosis

**Figure 1:**
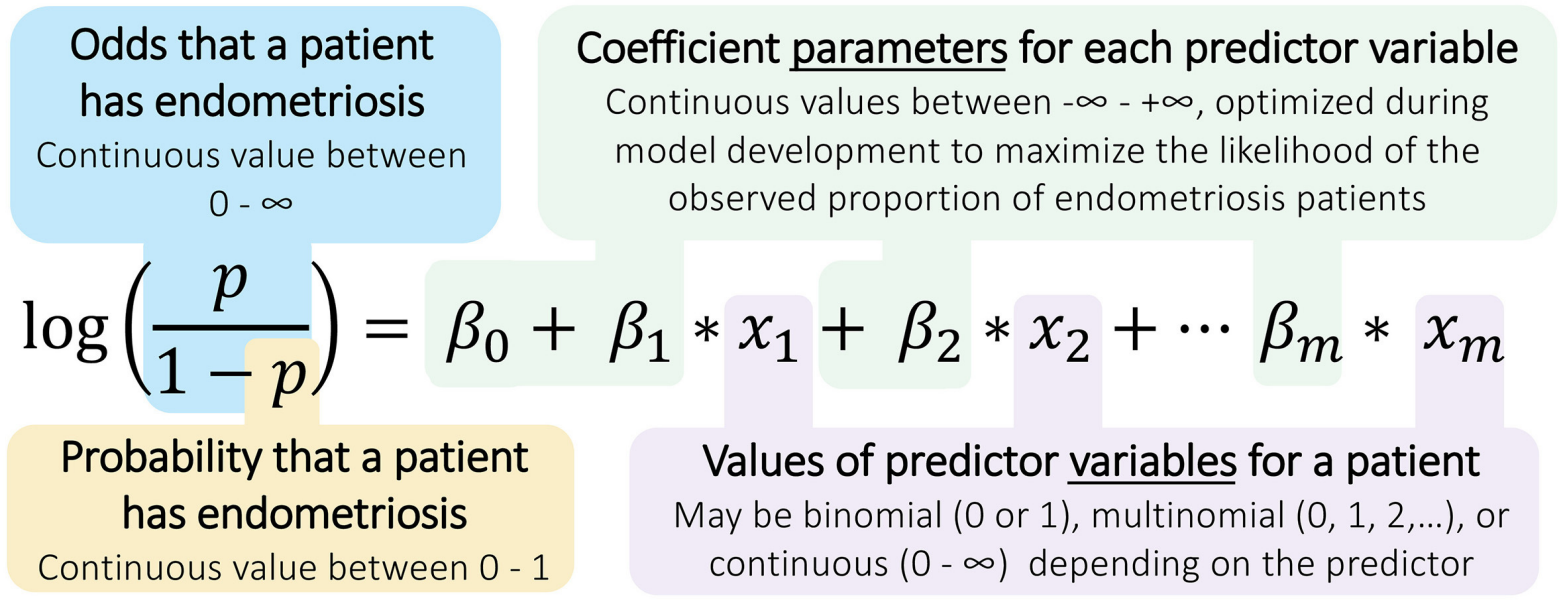
Structure of logistic regression models for diagnosing endometriosis. Logistic regressions calculate the odds and probability of a binary outcome (e.g., positive endometriosis diagnosis) using measurements taken across several predictor variables (e.g., patient observations). The model parameters, the β coefficients, are identified by applying the logistic regression model to a many-patient data set for whom the outcomes are known, and these coefficients can then be used with new patient data to predict the likelihood of endometriosis in that patient.

A logistic regression model estimates the probability of a binary outcome, such as having or not having endometriosis, using a set of independent observations about a patient as predictors (33). Regression models include components (Figure 1), in the form of these predictor variables, but they are not modeled as having any *spatial* or *temporal* relationships with one another; hence, these models are not considered mechanism-based (Table 1).

In medicine, logistic regression modeling is commonly applied to establish clinical scales, to identify risk factors for a disease, and to develop recommendations for treatment. For endometriosis, findings from logistic regression and related modeling have been cited as evidence for diagnosis guidelines [e.g., guidelines in (5) reference modeling in (34, 35)]. Specifically, researchers have used regression modeling to predict endometriosis from symptoms and medical history alone, blood tests alone, imaging alone, and a combination of these data sources (15).

Logistic regression does not require extensive prior knowledge of the mechanistic underpinnings of the disease, which can be difficult to ascertain. Instead, these models are entirely data-driven, using patient data that includes their known outcomes (e.g., diagnosis result) to predict the likely outcomes for other patients. Given sufficient data, logistic regression can identify the key elements that are predictive of endometriosis.

In this section, we will outline key points in creating a logistic regression model, using modeling by Nnoaham et al. (25) to illustrate these points. We discuss this study because it is one of the largest efforts so far to develop a non-surgical diagnosis for endometriosis, including more than 1,000 patients from 19 hospitals in 13 countries. The considerations detailed here will serve as a comparison point in later sections, where models are increasingly mechanism-based.

### Example: Logistic Regression Modeling to Identify Predictors of Endometriosis

**Figure 2:**
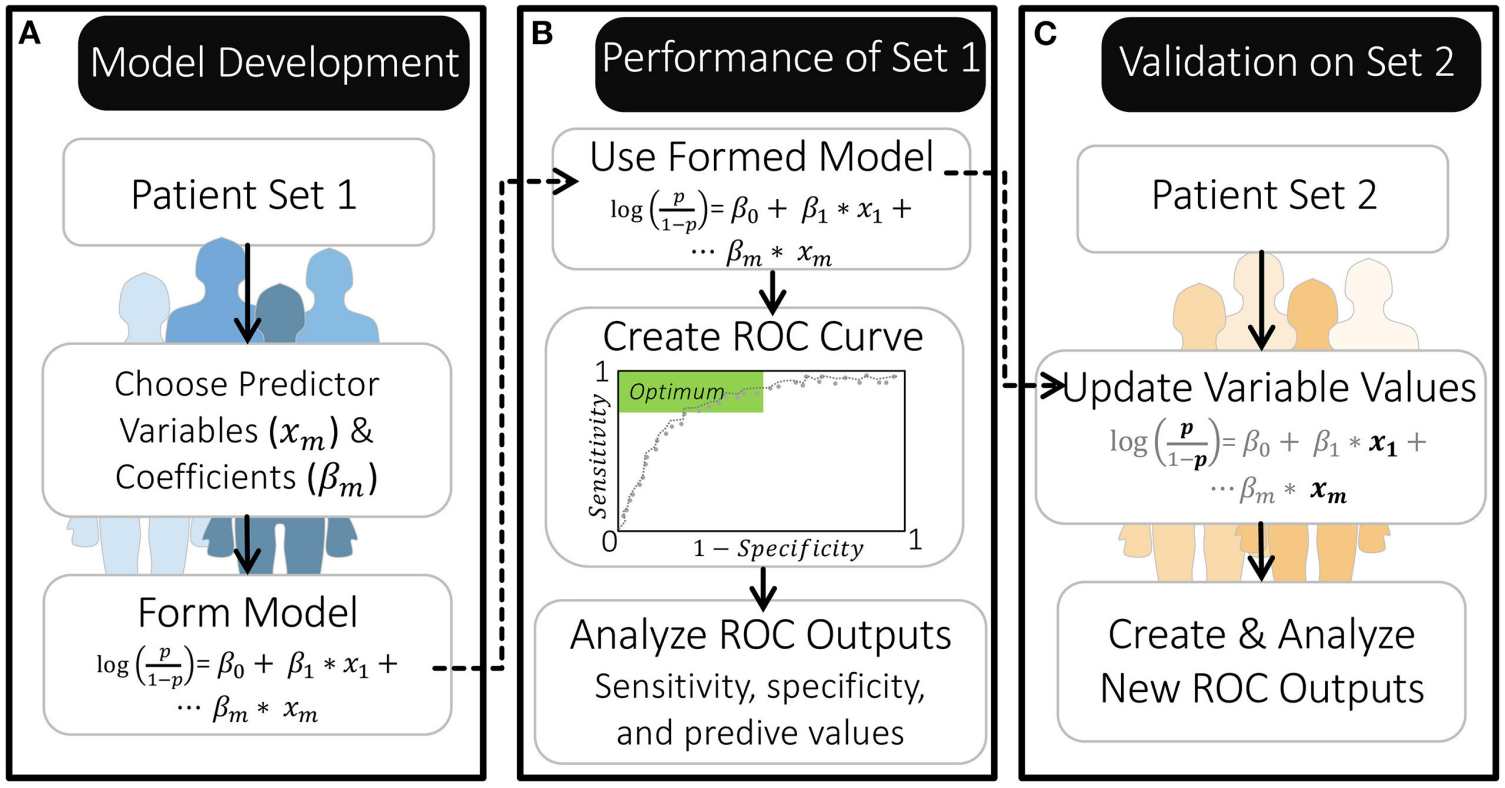
Overview of model development and validation by Nnoaham et al. (25). **(A)** The authors created their models using a set of 771 patients. **(B)** They then evaluated the performance of this model using a ROC curve to identify probability thresholds for classification that produce a specificity and sensitivity within the optimal range. **(C)** They further validated the model by first updating it with new predictor variable values for a separate set of 625 patients (leaving the β coefficients as they were) and then creating a new ROC curve.

As part of the Women's Health Symptom Survey study in 2012, Nnoaham et al. (25) developed and validated symptom-based predictive models to predict the probability of a patient having endometriosis prior to any diagnostic surgery (Figure 2). The patients in their study all suffered from pelvic pain and/or infertility and answered over 200 questions, detailing their demographics, medical history, and symptoms. The effect of including any preoperative ultrasound data was also explored (25).

The authors used logistic regression to calculate the probability that a patient would visually be diagnosed with endometriosis at laparoscopy based on a combination of the patient observations. The authors also calculated the probability of finding “moderate” to “severe” endometriosis, according to the revised American Society for Reproductive Medicine classification system (r-ASRM stages III-IV) (25).

#### Model Scope

For logistic regression models, researchers identify and include only the strongest predictor variables. Although models with many predictor variables may appear more accurate in fitting the training data, they can struggle to predict outcomes for new patients. To avoid this overfitting, researchers narrow the number of predictor variables included in their model, ideally having at least 10 patients for each predictor variable included (33).

Nnoaham et al. (25) identified which of the 200+ patient characteristics to include as predictor variables in their model by first grouping clinically-related predictors and then iteratively removing the predictor(s) in each group with the least significant association with endometriosis. Each of these reduced predictor groups were then combined, and the process was continued until each of their models included 18–25 predictor variables (i.e., one predictor variable for every 30–43 patients in their first patient set) (Figure 2A). These predictor variables had differing influence on the model's odds prediction, both in terms of sign and magnitude, which was reflected by their estimated regression coefficients (β). This approach to selecting model variables allowed the authors to minimize redundancy in predictor variables while maximizing how well the reduced model fit the data. Importantly, this process of forming model equations was primarily data-driven; meaning, mechanistic knowledge of how variables interact or contribute to disease was not used in selecting model variables or parameters (regression coefficients).

#### Data Usage

Logistic regression models are typically created using data from one study. If multiple studies are modeled, these studies must measure the model predictor variables and outcomes in a similar manner.

In constructing these models, Nnoaham et al. (25) used data from the Women's Health Symptom Survey study. As part of model development, all 1,396 patients in this study completed the same survey prior to their diagnostic surgery. This survey could capture a wide range of the patients' experiences, including predictor variables that were linear (e.g., age, average cycle length, menstrual flow) and categorical (e.g., ethnicity). Importantly, it was necessary for patients across the 19 hospitals to undergo the same assessment for the modelers to form a single estimate of the model parameters (the regression coefficients) that could predict the outcome (the diagnosis result) for all patients.

#### Clinical Impact

Through creating logistic regression models, researchers can identify a combination of characteristics that are highly predictive of a disease or treatment outcome. Clinicians can then use these findings to motivate further actions for patients with these characteristics. Hence, regression modeling aims to aid in the development of a less invasive diagnostic that correctly predicts endometriosis in those that have it (i.e. has a “high sensitivity”). Correctly identifying non-endometriosis patients (“specificity”) is also important—although less so if using this diagnostic to prioritize patients with subfertility for laparoscopic surgery, since laparoscopy can also identify other factors affecting fertility (36).

By constructing their model on one patient population, and evaluating it on a second, Nnoaham et al. (25) could assess how well their models would perform if applied to new patients. To evaluate their models, Nnoaham et al. (25) generated ROC curves (Figures 2B,C, Box 1) and found that their best model for diagnosing r-ASRM stage III-IV endometriosis achieved a sensitivity of 82.3% and specificity of 75.8% for their second set of patients. This sensitivity and specificity are sufficient if this predictive model is applied to develop recommendations for performing surgery to diagnose and treat endometriosis earlier—which is the usage that Nnoaham et al. (25) advocates for. This sensitivity would be insufficient if these model predictions were to be considered as exclusion criteria for diagnostic surgery or treatment, as ~18% of endometriosis patients would be missed.

Box 1 **Sensitivity, Specificity, and ROC.**

A model is assessed by creating a receiver operating characteristics (ROC) curve. The ROC curve shows the values for the true positive rate (“sensitivity”) vs. the false positive rate (1-“specificity”) at every possible probability threshold for classification (37).

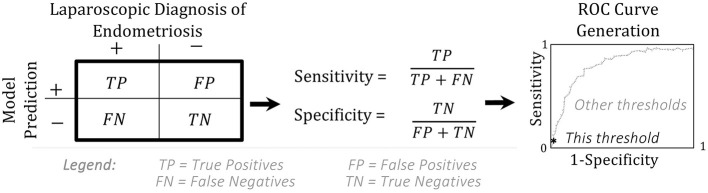

### Summary

As shown here, regression models serve as valuable tools for identifying patient characteristics that can predict disease or treatment outcomes. Importantly, this modeling does not explain the “how” in this association, as in: “how do these patient characteristics contribute to endometriosis and treatment outcomes?” To answer this question, researchers must model the mechanisms by which components within the system affect each other.

## Treating Endometriosis—Pharmacokinetic and Pharmacodynamic (PK-PD) Modeling

### Motivation for PK-PD Modeling

Medicinal approaches for treating endometriosis primarily aim to manage symptoms but have limited efficacy, with symptoms often recurring once a patient stops treatment (38). As a first-line therapy, many patients presenting with a combination of chronic pelvic pain or pain during menstruation, sex, or urination will take medications such as NSAIDS and hormonal contraceptives (38). For those with persistent pain and confirmed endometriosis, therapeutic options can include gonadotropin-releasing hormone (GnRH) analogs and aromatase inhibitors (5). These second- and third-line therapies are effective in treating chronic pelvic pain through suppressing estrogen, thereby inhibiting the growth and survival of endometriosis lesions (8, 39). However, GnRH analogs and aromatase inhibitors can be associated with severe hypoestrogenic effects, such as decreases in bone mineral density (38). Emerging clinical trials aim to identify novel therapeutic strategies for treating endometriosis with increased safety through applying an array of pharmacokinetic (PK) and pharmacodynamic (PD) modeling approaches. Here, PK modeling is applied to answer the question: How much drug will a patient be exposed to over time? PD modeling then considers: As drug exposure varies, how much of a physiological response can be expected?

### Use of PK-PD Modeling to Treat Endometriosis

**Figure 3:**
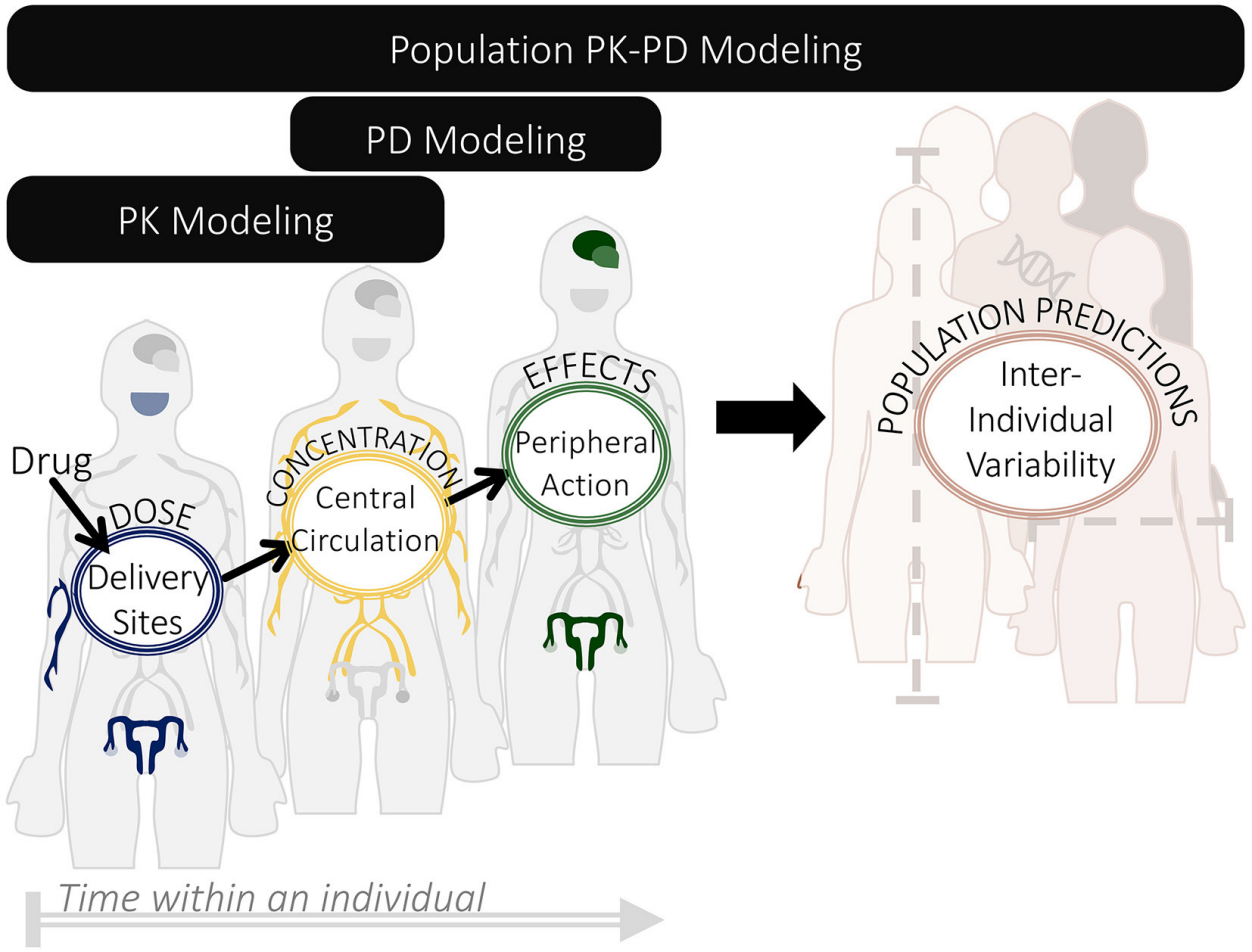
Relationship between pharmacokinetic (PK), pharmacodynamic (PD), and population PK-PD modeling. These three modeling modalities can be used to make predictions about treatment from drug dosing to resulting effects, on an individual and population scale.

Although treatments for endometriosis are monitored in circulating blood (“centrally”) many drugs are delivered to or act throughout peripheral sites. To predict drug exposure or efficacy, we need a way to connect these sites. Pharmacokinetic modeling connects these central and peripheral sites through equations that predict the concentration of a drug as it is absorbed, distributed throughout the body, and eliminated via metabolism or excretion. Pharmacodynamic modeling uses these estimated and monitored drug levels to predict the onset, duration, and intensity of response to that drug (40). Population PK-PD models incorporate variability in select model parameters based on differences between patients (e.g., body mass, age, and genetic background), allowing for simulations of larger virtual populations to better inform recommendations (Figure 3).

**Figure 4:**
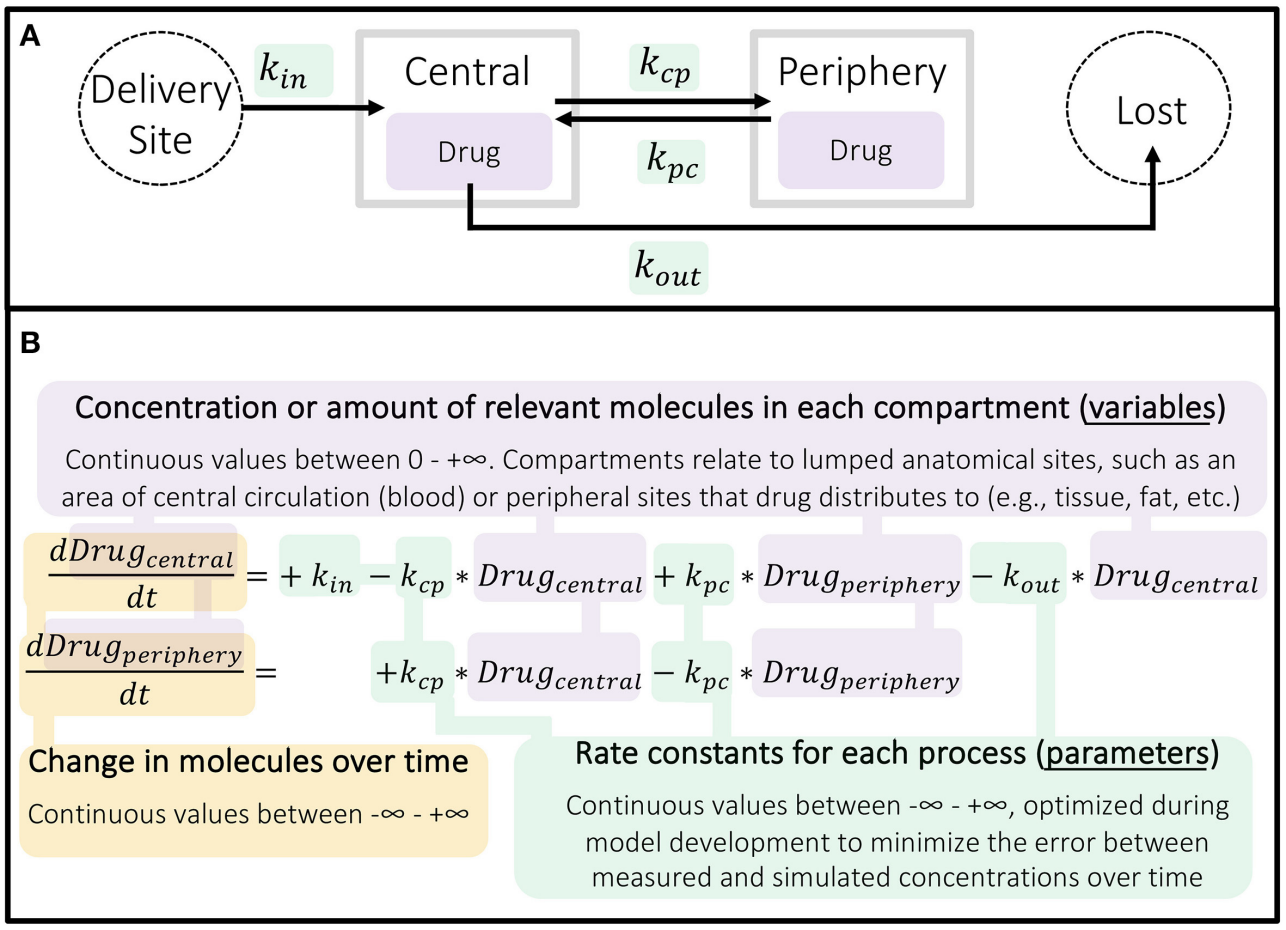
Structure of basic two-compartment pharmacokinetic model. **(A)** Schematic of continuous processes represented in a two-compartment model. **(B)** The two ordinary differential equations (ODEs) used here describe the rate of change in concentration of the drug in the central and peripheral compartments over time as a result of these processes occurring. PK models can be more or less complex, with different compartments and processes included as needed to fully describe the drug being investigated in the simplest reasonable form.

The commonly applied two-compartment (central and peripheral) pharmacokinetic model incorporates all five elements of mechanism (Table 1)—modeling how a *component* (drug) moves through *space* and *time* in the *context* of drug dosing in order to predict *phenomena*, such as drug efficacy or toxic effects. Unlike regression modeling (Figure 1), pharmacokinetic models are composed of differential equations, where the variables are the concentration (or amount) of each component and the parameters are the rate constants representing how fast reactions and transitions between components and compartments occur (Figure 4).

Because of this structure and level of mechanistic detail, pharmacological models are ideal for simulating and comparing different dose amounts, regimens, and delivery sites for endometriosis therapies under development. By developing PK-PD models with additional mechanistic detail, researchers have been able to identify endometriosis patients with a genetic favorability for a GnRH antagonist (41), predict changes in bone mineral density following long-term GnRH antagonist treatment of endometriosis (42), and interrogate the role of chosen delivery method in the efficacy of combination progestin therapies (43).

As we discuss the unique considerations in population PK-PD models, we will use (26) as an example. This study by Reinecke et al. applied PK-PD modeling to select doses to be used in phase 2 of a clinical trial for an endometriosis therapy. In addition to modeling the distribution of the therapy throughout the body, this model predicted the influence of endogenous proteins on drug efficacy and adverse events in patients. This study is also of interest because of its application of multiscale data—ranging from *in vitro* experiments to animal experiments and previous phase 1 studies—to select the equations and parameters for this model.

### Example: PK-PD Modeling to Design Clinical Trials

In 2017, Reinecke et al. (26) used population PK-PD modeling to guide the development of an intravaginal ring (IVR), delivering the aromatase inhibitor anastrozole (ATZ) and the progestin levonorgestrel (LNG) for long-term, localized treatment of endometriosis and associated pain (26). This new approach to treating endometriosis targets estrogen production in endometriotic lesions through local inhibition of aromatase, thereby minimizing systemic hypoestrogenic effects. This therapy also includes a progestin to provide contraception because ATZ is a teratogen (44).

Population PK-PD modeling was used to identify doses that would achieve therapeutic levels of ATZ and LNG while minimizing the risk of ovarian cysts in a phase 2 clinical trial (EudraCT 2013-005090-53; NCT02203331) (26). These PK-PD models use ordinary differential equations (ODEs) to predict the levels of drugs in the body over time and the associated risk of developing ovarian cysts.

**Figure 5:**
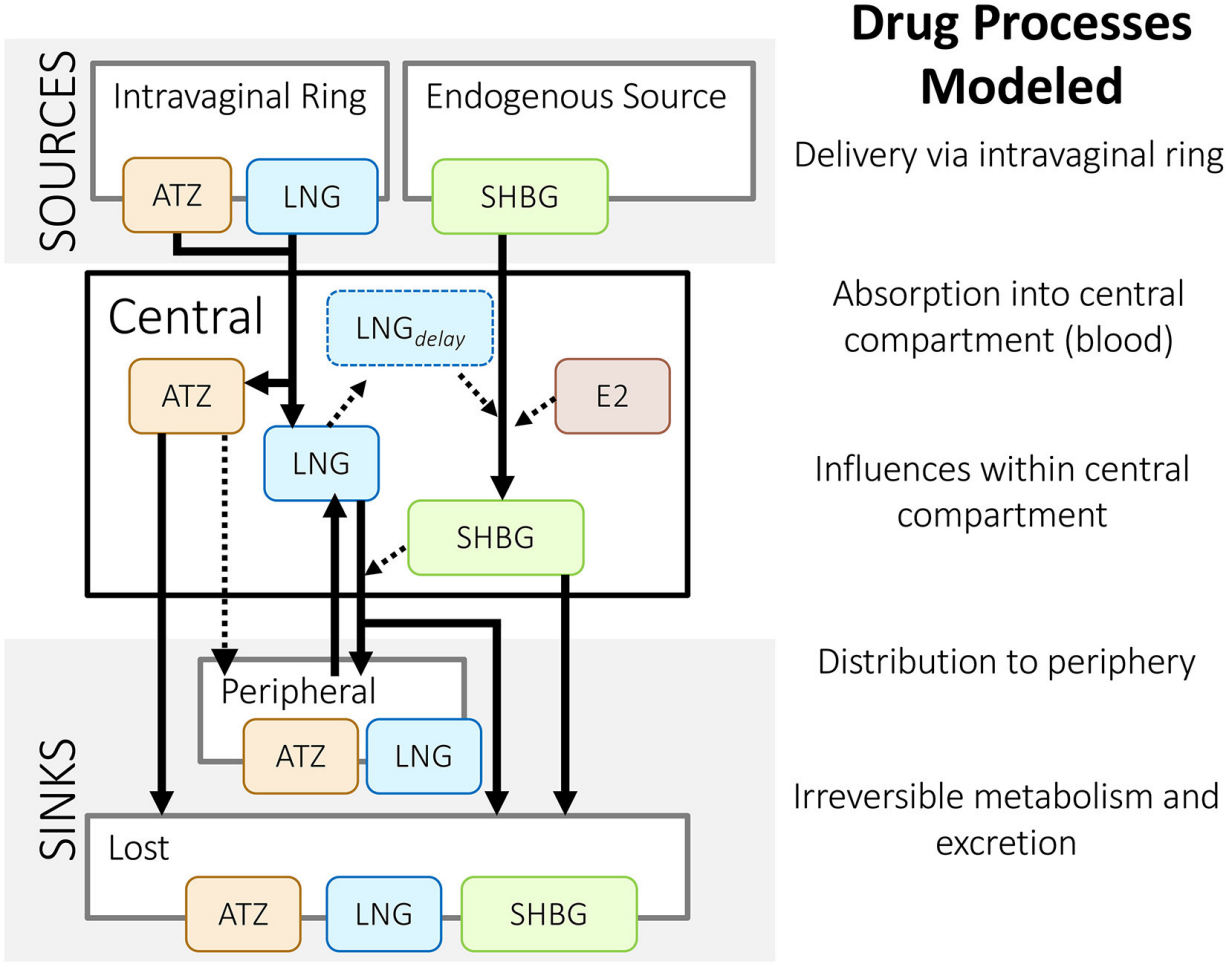
Structure of Reinecke et al.'s (26) population pharmacokinetic models for an intravaginal ring that delivers anastrozole (ATZ) and levonorgestrel (LNG). These models include the influence of estradiol (E_2_) and LNG on sex hormone binding globulin (SHBG), and vice versa. Solid lines represent a mass flow; dashed lines represent an indirect influence – as described in Reinecke et al. (26).

Each drug was modeled using a two-compartment model as a basis (Figure 4). Using data from *in vitro* and animal studies alongside their mechanistic understanding of the system, Reinecke et al. (26) amended the ATZ and LNG base models to more closely match the outcomes of a phase 1 clinical study in humans (EudraCT 2011-005620-18). As a result, these models included delivery via an intravaginal ring. In addition, since LNG predominantly binds to and influences the production of sex hormone binding globulin (SHBG) in serum, the LNG model also included the influence of LNG on SHBG (and vice versa) and the additional influence of circulating estradiol (E2) on SHBG (Figure 5).

#### Model Scope

In contrast to regression models, where deciding the scope was fully data-driven, the equations in pharmacological models can be developed in both a mechanism-based and data-driven manner. In constructing a pharmacokinetic model, researchers can consider the biology of a drug and its interactions within the body to better understand and improve upon the therapy.

For example, Reinecke et al. (26) chose to include sex hormone binding globulin (SHBG), a circulating protein that binds the delivered LNG and endogenous estradiol (E2). LNG and E2 were both modeled as indirect influences on the rate that SHBG is produced (Box 2). Inclusion of these molecules allowed the researchers to explore the role of SHBG in contraceptive efficacy and ovarian cyst formation. As a result, the simulations were able to capture fluctuations that appeared in clinical measurements. By including E2 and SHBG in their model, Reinecke et al. (26) could also explore the influence of observed inter-individual variability, such as variability in SHBG and E2 baseline levels, as they made population-level predictions.

Box 2 **Differential equation for SHBG from Reinecke et al. (26) model.**

The model for SHBG in the blood over time [*SHBG*(*t*)] includes terms that affect its production and loss, which have rate constants *k*_*in*_ and *k*_*out*_, respectively. The production term is influenced by delayed inhibition by LNG and induction by E2, which scale rate constant *k*_*in*_ by a factor of −*r*_*i*_ and +*r*_*s*_, respectively. The loss term is linearly proportional to the level of SHBG in the blood.

$$\begin{array}{l} \frac{d}{dt}SHBG\left(t\right)=\;+k_{in}\;*\;\left(1-r_{i}\;*\;LNG_{delay}\left(t\right)+r_{s}\;*\;E2\right)-\;k_{out}\;*\;SHBG\;\left(t\right) \end{array}$$

#### Data Usage

Pharmacological models are created using data that characterize the mechanisms contributing to drug delivery and response. Unlike regression modeling, this data can come from multiple independent studies that assess different aspects of the biological system. Hence, processes affecting a drug can be evaluated in isolation prior to being incorporated into a pharmacokinetic model.

Reinecke et al. (26) used data from *in vitro* experiments measuring daily release from an intravaginal ring to create and parametrize equations describing delivery via the intravaginal ring, specifically. In using this data, they assumed that a ring under their bench-top conditions releases drug in a similar manner to a ring within a vagina, which they support using evidence from a preclinical study conducted in cynomolgus monkeys. This *in vitro* data was used in combination with phase 1 clinical data, which included plasma drug concentrations and drug remaining in the ring at the end of treatment, to create and parametrize their model. As a result, this model can predict multiple patient outcomes over time, including: the level of drug in the intravaginal ring, serum concentrations of the delivered drugs (LNG and ATZ), as well as the levels of influencing molecules (E2 and SHBG).

#### Clinical Impact

Clinical researchers use pharmacokinetic modeling to explore drug dosing in populations or treatment groups. In addition, by integrating these PK models with pharmacodynamic (PD) models, clinical researchers can predict drug effects and identify predictors for adverse events.

**Figure 6:**
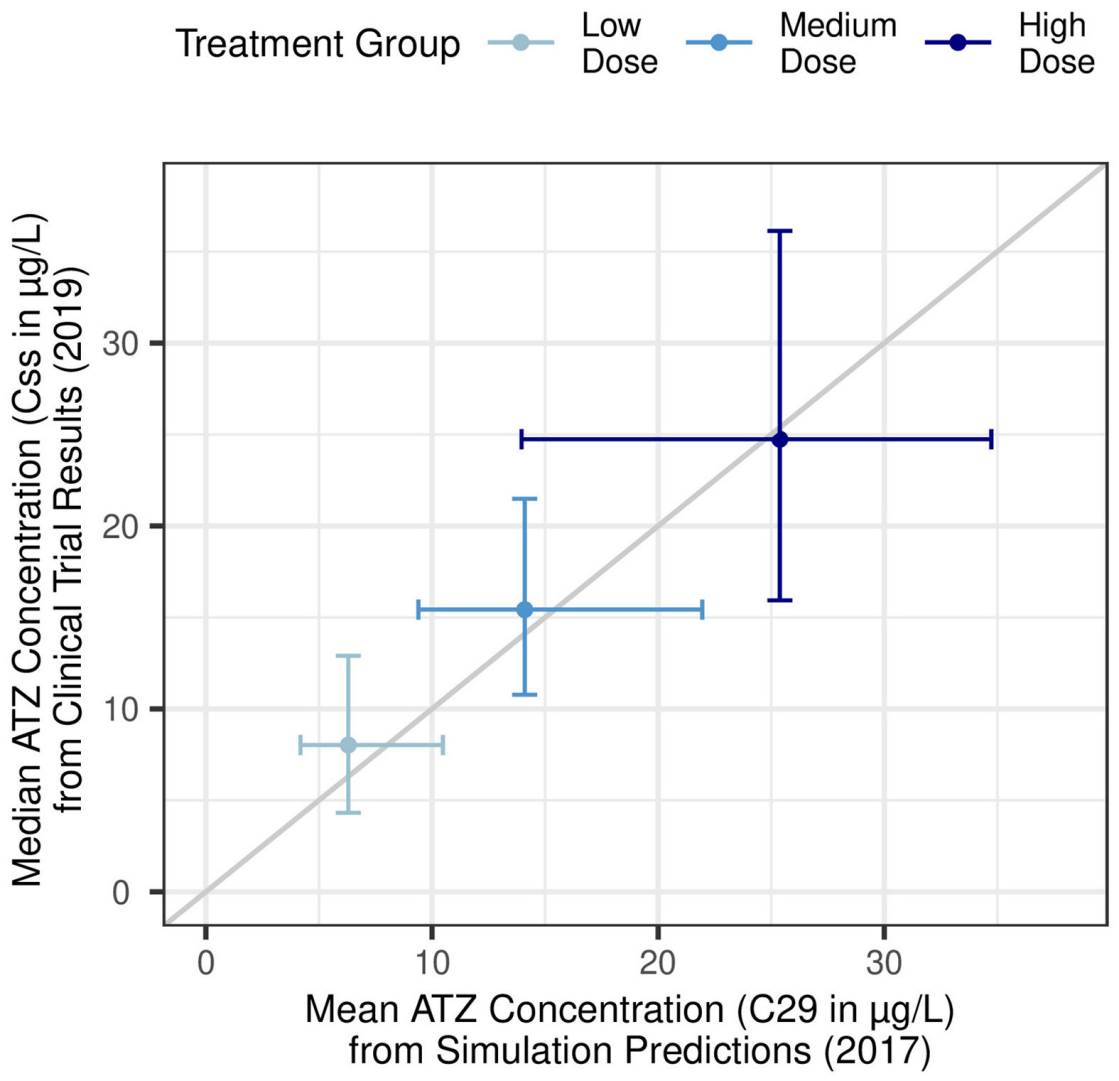
Agreement between pharmacokinetic simulation predictions and subsequent clinical trial results. X-axis: Simulation predictions for mean plasma ATZ concentrations 28 days following ring placement (C_29_) in low-dose (290 μg/day), medium-dose (630 μg/day), and high-dose (1,080 μg/day) treatment groups. Horizontal error bars represent the 5th and 95th percentiles. Adapted from Reinecke et al. (26). Y-axis: Observed median plasma ATZ concentration as average of measurements taken 28, 56, and 84 days following first ring placement (C_ss_) in low-dose (300 μg/day), medium-dose (600 μg/day), and high-dose (1,050 μg/day) treatment groups from a phase 2b clinical trial. Vertical error bars represent the 10th to 90th percentile. Adapted from Nave et al. (45).

Once Reinecke et al. (26) confirmed their simulations matched the phase 1 (EudraCT 2011-005620-18) results for three intravaginal ring formulations, they used their models to simulate additional doses of ATZ and LNG. These researchers were then able to identify three additional ATZ doses for a phase 2 trial. They used modeling to identify doses that could achieve the minimum effective concentration for all patients, while having minimal overlap between treatment groups, thereby maximizing the potential insight gained. Remarkably, the predictions from Reinecke et al. (26) closely matched results from a subsequent phase 2 study in endometriosis patients (45) (Figure 6).

Furthermore, Reinecke et al. (26) created a PK-PD model in order to predict the effect of LNG and ATZ exposure on ovarian cyst formation. They compared the predicted probability of developing ovarian follicles ≥ 30 mm between several PD models, which varied in the relative influence of ATZ and LNG exposure. They selected the best model by comparing the predicted probabilities to the observed fraction of patients with enlarged follicles found during ultrasound. In the end, they found that increasing unbound LNG levels are more predictive of large follicle formation than increasing ATZ levels. This model could be used to predict the risk of developing ovarian cysts for the doses they were selecting for phase 2 of their clinical trial.

### Summary

As shown through the Reinecke et al. (26) example, population PK-PD modeling can be useful in deciding study treatments, simulating population heterogeneity, and predicting treatment response using information from *in vitro*, animal, and human studies. These insights inform the design of clinical trials and, ultimately, how a drug is used to treat disease. Differing from the logistic regression model, PK models predict changes in component concentrations over time, painting a dynamic picture of the system. Although the base two-compartment model is quick to create and often resembles typical drug exposure, this approach limits the questions researchers can address through modeling. As such, modelers often choose to include more mechanistic detail in their PK model and incorporate population variability in parameter values, as Reinecke et al. (26) has done.

## Modulating the Menstrual Cycle—Quantitative Systems Pharmacology (QSP) Models

### Motivation for QSP Modeling

Endometriosis treatment is complicated by the systemic effects of estrogens, gonadotropins, and related hormones throughout the menstrual cycle. Therefore, there is significant interest in understanding and predicting the effects of therapies that perturb the cycle, such as gonadotropin-releasing hormone (GnRH) analogs, aromatase inhibitors, and progestins, on endometriosis and subfertility. To do so, mechanism-based systems biology models have been created using differential equations to describe systemic hormone fluctuations that occur during the menstrual cycle (31). Quantitative systems pharmacology (QSP) connects pharmacokinetic (PK) models of hormone-modulating therapies with models of those hormones and of endogenous protein signaling in order to further study the effect of these drugs on the body. In contrast to pharmacodynamic (PD) modeling, which predicts the change in magnitude of a physiologic response, QSP modeling allows us to consider: What are the underlying mechanisms contributing to a physiological response and how can they be best therapeutically targeted?

### Use of QSP Modeling to Develop Treatments for Endometriosis

Quantitative systems pharmacology (QSP) integrates systems biology approaches with both data-driven and mechanism-based computational techniques to understand and optimize therapies (28). Upon first glance, the structure of QSP models resembles that of PK-PD models, using differential equations to represent changes in proteins in the system over time (Figure 4). But while PK-PD models tend to be drug-centric (predicting the distribution and effects of exogenous compounds), QSP models also focus on processes endogenous to the body. QSP models thus allow us to answer questions that are more mechanism-focused than typical PK-PD models, because they model the influence of molecules from the sub-cellular to multi-organ levels, thereby including more *components* interacting over more *spatial* and *temporal* scales (Table 1).

QSP models have been created to explore the effects of therapies on protein signaling that impacts endometriosis. For example, Riggs et al. (46) expanded upon a mechanism-based model of bone remodeling (47) to study the effects of therapeutic estrogen-suppression to treat endometriosis (46). Importantly, Riggs et al. (46) combined their QSP model with a logistic regression model to assess how well patients' estrogen levels could predict their endometriosis-related pain severity—illustrating how the models discussed in this review can be used in harmony (46). In addition, QSP models have been used to predict *in vivo* treatment outcomes from *in vitro* systems, such as novel microphysiological systems that include the endometrium (48).

Röblitz et al. (27) created a QSP model of hormone cycling to aid in the development of GnRH analog therapies. GnRH analogs are critical in treating several conditions, including: cancers, uterine fibroids, and infertility (27). Although this study was not focused on endometriosis, we are discussing it because they model the GnRH antagonist, cetrorelix, which is used to treat endometriosis (38). Here, we will explore how these researchers integrated a highly mechanistic model of the menstrual cycle with pharmacokinetic models of GnRH analogs in order to compare treatments.

### Example: QSP Modeling to Guide Menstrual Cycle Modulation

**Figure 7:**
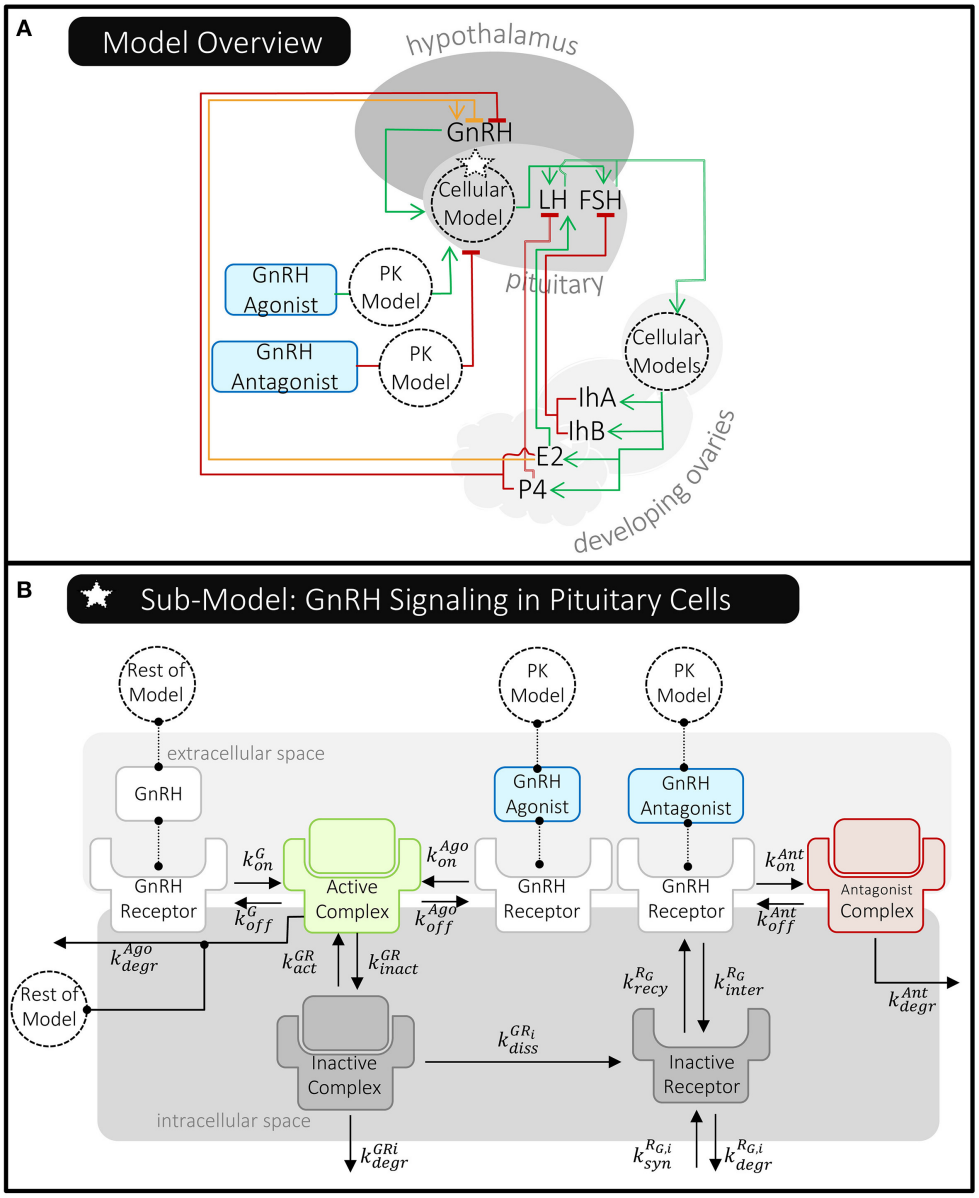
Overview of Röblitz et al.'s (27) quantitative systems pharmacology model of the menstrual cycle and gonadotropin-releasing hormone (GnRH) therapies. **(A)** This schematic shows where molecules are produced and whether they stimulate (green line/arrow head), inhibit (red line/flat head), or have a mixed effect (orange line/both) on the production of other molecules in this model. The dotted circles labeled “Cellular Model(s)” represent processes affecting pituitary GnRH receptors and ovarian LH/FSH receptors that have been modeled in detail. The delivery of GnRH agonist and antagonist are modeled using PK models that feed into the pituitary cellular model. **(B)** The pituitary cellular model is summarized here. Each reaction has a unique reaction rate constant (*k*) that can depend on the receptor state (e.g., whether it's internalized or the specific molecule it's bound to). For simplicity, reactions involving an active complex have just been shown once; however, the rates of these processes do depend on the receptors' states, as described in Röblitz et al. (27).

Röblitz et al. (27) modeled key hormones that travel and signal between the brain, ovaries, and the blood (Figure 7A). In the body, and specifically included in the model, GnRH is formed in the hypothalamus and transported to the pituitary gland where it stimulates the release of the gonadotropins, luteinizing hormone (LH) and follicle stimulating hormone (FSH), into the bloodstream. LH and FSH exert their effects on processes in the ovaries, which include follicular development, ovulation, and the development of the corpus luteum. Through these processes, the production and release of progesterone (P4), estradiol (E2), and inhibins A and B (IhA, IhB) are regulated. These circulating hormones signal back to the hypothalamus and pituitary, affecting the formation and release of GnRH, LH, and FSH.

Röblitz et al. (27) also created pharmacokinetic models of GnRH analog delivery to connect to these highly mechanistic models of the menstrual cycle. The delivery of GnRH agonist and antagonist are modeled using a one- and two- compartment PK model, respectively—similar to those previously described (Figure 4). Röblitz et al. (27) incorporated the pharmacokinetic model of the GnRH agonist, nafarelin, by modeling the drug in the central compartment (circulating blood) as being able to bind to and activate GnRH receptors, as natural GnRH does. In contrast, the GnRH antagonist, cetrorelix, is modeled as being able to bind to but not activate GnRH receptors (Figure 7B). In this way, the administered drugs either act alongside or compete with GnRH, thereby affecting the level of GnRH receptors available to activate downstream signaling.

#### Model Scope

How much physiological detail to include in a mechanistic model is often a balance of the questions being explored and the computational resources and data available.

This delicate balance is illustrated in comparing Röblitz et al. (27) to Reinecke et al. (26). As discussed in the previous section, Reinecke et al. (26) was primarily interested in predicting drug exposure over time and how that affected the odds of ovarian cyst development. As such, drug-protein interactions were primarily modeled as indirect influences, either increasing or decreasing the level of free drug in central circulation (Figure 5). In contrast, since Röblitz et al. (27) sought to predict both drug exposure and the effects on signaling throughout the menstrual cycle, these researchers more directly modeled the physiologic processes that together impact the delivery and effect of GnRH analogs. This included hormone-receptor interactions in the brain and ovaries, as well as ovarian follicle maturation (Figure 7). In contrast to how Reinecke et al. (26) models the effects of E2 and LNG on the level of SHBG (Box 2), Röblitz et al. (27) uses mass action kinetics to represent each interaction and process that alters the level of GnRH receptor on the cell surface (Box 3). By creating similar equations for the processes affecting GnRH, other hormones, and their receptors, Röblitz et al. (27) could accurately predict the timing of ovulation under various treatment scenarios.

Box 3 **Structure of differential equation for GnRH receptor from Röblitz et al. (27).**

This sub-model uses mass action kinetics to predict cumulative effect of each process on the level of free (unbound) GnRH receptor on the surface of pituitary cells over time [*R*_*G,a*_ (*t*)]. These processes include (listed in order they appear in this equation): binding and unbinding to endogenous GnRH, receptor internalization and recycling—from and to the cell surface, and receptor binding and unbinding to GnRH agonist (“Ago”) and antagonist (“Ant”). The rate constant for each reversible reaction is represented by each “*k*” term below, which are also shown in Figure 7B. Refer to Röblitz et al. (27) for full details and equations.

$$\begin{array}{l} \frac{d}{dt}R_{G,a}(t)=\;-\;k_{on}^{G}\;*\;G(t)\;*\;R_{G,a}(t)+k_{off}^{G}\;*\;GR(t)\\ \;-\;k_{inter}^{R_{G}}\;*\;R_{G,a}\;(t)+k_{recy}^{R_{G}}\;*\;R_{G,i}\;(t)\\ \;-\;k_{on}^{Ago}\;*\;SF_{Ago}\;*\;Ago_{c}(t)\;*\;R_{G,a}\;(t)+k_{off}^{Ago}\;*\;AgoR(t)\\ \;-\;k_{on}^{Ant}\;*\;SF_{Ant}\;*\;Ant_{c}(t)\;*\;R_{G,a}\;(t)+k_{off}^{Ant}\;*\;AntR(t) \end{array}$$

Although Röblitz et al. (27) models major hormonal and physiological components of the menstrual cycle in significant detail, they do limit their model scope to minimize computational load. For example, as Röblitz et al. (27) created equations to model GnRH signaling, they avoided operating on the small time scales (minutes) that had been previously used to model GnRH pulsations. Röblitz et al. (27) acknowledges that not including a more detailed GnRH pulsing model may be limiting their model's accuracy. However, this reduction in model parameters may also be allowing for a more robust model—sacrificing a model's ability to perfectly predict one component (or scenario) often produces a model that is better able to predict many components (and scenarios).

#### Data Usage

Similar to PK-PD models, QSP models are created using data from independent studies to characterize drug delivery and effects. However, these researchers must use additional data to model biological mechanisms on multiple scales, even more-so than typical PK-PD models.

Like pharmacokinetic approaches, Röblitz et al. (27) created and parametrized their model of hormone cycling (without GnRH treatment) using daily hormone measurements taken from 12 people with normal menstrual cycles. However, Röblitz et al. (27) connected these models to cellular models of LH and FSH in the ovaries, as well as GnRH in gonadotropic cells of the pituitary (Figure 7B). At the cellular level, the rate of GnRH receptor binding and trafficking were estimated using data from an earlier model by Blum et al. (49)—this model estimated these reaction rates using experimental measurements of gonadotropes in culture. Through applying both clinical and *in vitro* data, Röblitz et al. (27) was able to not only track the levels of cycling hormones (e.g., LH, FSH, E2, P4, etc.) over time, but they could also predict the concentrations of proteins that aren't currently measured in patients (e.g., LH-receptor and GnRH-receptor complexes). This allows for a multi-scale understanding of how treatments are affecting patients and can be further analyzed to identify alternative therapeutic approaches.

#### Clinical Impact

QSP models include more biological components, such as endogenous protein or hormone networks, than a typical PK-PD model. Because of this, clinical researchers often use QSP models to compare multiple therapeutic strategies and diseases.

The Röblitz et al. (27) model was successful in simulating not only the levels of each drug over time, but also the resulting fluctuations in patients' hormone levels. This produced a versatile model that could be used by clinical researchers to compare the effects of dosing GnRH agonists and antagonists on hormone cycling and the resulting effects on ovulation. As one example of this utility: Through modeling, Röblitz et al. (27) found that the GnRH antagonist, cetrorelix, delays ovulation in a manner that is highly dependent on each patient's drug clearance rate. This suggests that if a patient's plasma drug concentrations are monitored in the first day of dosing, then a clinician may be able to more accurately predict when ovulation will occur and when subsequent doses may be necessary.

Furthermore, because the Röblitz et al. (27) model includes both the direct targets of GnRH analogs (e.g., bound receptors) and the indirect targets (e.g., developing follicles, circulating hormones), this model can make predictions about system behavior when anything in the model is perturbed. For example, endometriosis is characterized as a hyper-estrogenic state. Because estradiol is included in the model, this model could be used to examine how elevated estradiol affects ovulation and signaling within the menstrual cycle. In addition, exogenous molecules that affect the hormones and receptors already present could be explored with minimal adjustments or additions to the model.

### Summary

The Röblitz et al. (27) model combines approaches from traditional PK-PD models with a highly mechanistic, QSP model to compare the effects of GnRH agonists and antagonists on people with normal menstrual cycles. This allows their model to efficiently predict clinical measures while supplying more insight into the biological processes affected by perturbations caused by disease or treatment than a PK-PD model alone could. Importantly, QSP models can be adapted to study different disease or treatment cases. This may involve applying the model to a new set of patients and/or adapting the model to include additional disease-related biological processes, such as in Riggs et al. (46). Ultimately, these highly mechanistic, systems biology models aim to expand (in both number and complexity) the biological questions researchers can explore.

## Conclusion

### Benefits and Limitations of Each Computational Modeling Approach

In this review, we've explored three mathematical modeling techniques that have been applied to improve endometriosis diagnosis and treatment: regression, pharmaco-kinetics/dynamics (PK-PD), and quantitative systems pharmacology (QSP). Below, we'll summarize the benefits and challenges of each modeling approach and outline opportunities for future modeling of endometriosis.

Regression models represent a data-driven approach; meaning, they can be created without needing to start with a detailed mechanistic understanding of the system. As a result, regression models excel in identifying associations in data (e.g., which measured variables or combinations of variables are strong predictors of endometriosis or of clinical outcomes) without requiring advance knowledge of how these associations contribute to disease. However, these models are limited in their ability to explore the “how” in these associations.

PK±PD and QSP models both represent mechanism-based approaches that can be used to predict how biological factors will influence patient treatment. PK models are especially useful for deciding drug dosing in clinical studies. Although base compartmental PK models only predict the distribution of a drug throughout the body, researchers can add details about drug interactions within the body to the model (if that data is available). This leads to the creation of a more mechanistic PK-PD model. However, to better understand the role that endogenous pathways play on any disease and treatment, a QSP model is used.

QSP models closely resemble PK-PD models; however, QSP models add more focus on the biological mechanisms endogenous to the system. This leads to the inclusion of a wider range of experimental data to parametrize a QSP model (e.g., from molecular and cellular to tissue and multi-organ levels). As a result, QSP models can simulate changes within a biological system without any drug introduced—this is something PK models do not do. QSP models thereby become increasingly useful in interrogating the mechanisms underlying a drug response and contributing to disease. QSP models are also well-suited for comparing multiple disease and treatment scenarios. However, these models can be more time- and knowledge-intensive to create.

### Gaps in Modeling Endometriosis and Opportunities for Future Models

There are still many opportunities for the development and improvement of computational models to diagnose and treat endometriosis. Regression models need a large (many-patient) dataset across multiple clinical centers [such as in (25)] in order to have findings that can be generalized to other endometriosis patients. As discussed in previous reviews (15, 18), many studies on diagnostic indicators of endometriosis either have too few patients to be generalized or have yet to be validated with an independent patient population. Additionally, regression models predicting treatment outcomes are less common, so have not been discussed here. However, recent studies have used regression modeling to predict the efficacy of assisted reproductive technology and surgery on the fertility outcomes for endometriosis patients (50, 51).

The limitations of PK-PD studies often relate to the availability of sufficient data. How much data, and which data, is needed to model a therapy's delivery and effects will depend on the properties of that specific therapy. Models can be augmented with pre-clinical data and data from previous trials, as in Reinecke et al. (26). Additionally, there has been increased use of more mechanistic PK models, such as physiologically-based PK models, for investigating drug-drug interactions of therapies for endometriosis (42, 52). This could be due to the expanded tools for establishing, analyzing, and submitting these models for regulatory review (53, 54).

QSP models in general are a more recent approach. Several QSP models have been created to investigate the effects of hormone-modulating therapies on cell signaling in people with normal menstrual cycles and in people with polycystic ovary syndrome. So far, few of these models have directly modeled the effects of these therapies in endometriosis—with Riggs et al. (46) being one of the few. These researchers created a mechanism-based model to predict the effects of therapies on endometriosis symptoms and bone mineral density (46). As endometriosis is known to involve dysregulation in hormone, vascular, and immune signaling networks, there are several opportunities to use highly mechanistic computational modeling, such as QSP, to further our ability to understand, diagnose, and treat endometriosis.

For instance, the mechanism-based models of hormone signaling outlined in this review could be adapted to study the effects of hormones on endometrial tissue. One recent study has connected a hormone signaling model to a newly developed mechanistic model of endometrial changes during the menstrual cycle, including terms to represent growth, shedding, and blood vessel development (55). This and future studies can be used to explore the effects of endometriosis-associated hormone dysregulation on the endometrium.

Focusing on vascular and immune influences, researchers can adapt mechanism-based models of protein-signaling in blood vessel development (56) to study the impact of endometriosis lesions producing pro-angiogenic cytokines [e.g., VEGF, IL-1β, IL-6, IL-8, etc. (9)]. Additionally, mechanism-based, systems biology models can help us interrogate the interactions between endometrial and immune cells in endometriosis. Since endometriosis lesions and cancerous tumors share some immune and vascular abnormalities, cancer models may serve as a basis for this. For instance, macrophages are known to affect endometriosis lesions as they differentiate, secrete cytokines, and promote angiogenesis (13)—Mahlbacher et al. (57) have modeled these macrophage behaviors within cancerous tumors. Lastly, agent-based models (another mechanism-based approach) have been created to study signaling and development of epithelial tissues (58), such models can be adapted in order to investigate the functioning of healthy and endometriotic epithelia within organoid cultures.

Since each modeling approach yields distinct insight, data-driven and mechanism-based modeling can and have been used in concert to identify associations in biological data and interrogate the underlying mechanisms of disease, respectively. The harmony of these approaches was demonstrated as we discussed previous QSP models (27, 47). By using a multitude of computational modeling approaches, researchers can synthesize multiscale experimental and clinical data to identify predictors of endometriosis and design therapies. Furthermore, there are exciting opportunities for developing mechanism-based models to discern how disruptions in cell signaling affect immune, vascular, and hormone systems, and ultimately, contribute to endometriosis.

## Author Contributions

WM performed literature search for and wrote the final draft of this manuscript. AG wrote a section for the first draft of this manuscript. FM, WM, and AG contributed to the conceptualization of this manuscript, manuscript revision, read, and approved the submitted version. All authors contributed to the article and approved the submitted version.

## Funding

This study was supported by the National Science Foundation Graduate Research Fellowship Program under Grant No. DGE1746891 (to WM) and the National Institutes of Health Medical Scientist Training Program under Grant No. T32GM136577 (to AG).

## Conflict of Interest

The authors declare that the research was conducted in the absence of any commercial or financial relationships that could be construed as a potential conflict of interest.

## Publisher's Note

All claims expressed in this article are solely those of the authors and do not necessarily represent those of their affiliated organizations, or those of the publisher, the editors and the reviewers. Any product that may be evaluated in this article, or claim that may be made by its manufacturer, is not guaranteed or endorsed by the publisher.
